# CAcnesDB: a database of *Cutibacterium acnes* with integrated functional insights derived from multi-modal genome annotation

**DOI:** 10.1093/database/baag067

**Published:** 2026-11-10

**Authors:** Bandana Pandia, Sneha Vasudevan, Dheemanth Regati, Nihesh Rathod, Santhosh Sankar, R Sowdhamini, Nagasuma Chandra

**Affiliations:** Department of Biochemistry, Indian Institute of Science, Bangalore, Karnataka 560012, India; IISc Mathematics Initiative, Indian Institute of Science, Bengaluru, Karnataka 560012, India; National Centre for Biological Sciences, TIFR, GKVK Campus, Bellary Road, Bangalore 560065, India; Department of Electrical Communication Engineering, Indian Institute of Science, Bengaluru, Karnataka 560012, India; Department of Biochemistry, Indian Institute of Science, Bangalore, Karnataka 560012, India; National Centre for Biological Sciences, TIFR, GKVK Campus, Bellary Road, Bangalore 560065, India; Department of Biochemistry, Indian Institute of Science, Bangalore, Karnataka 560012, India; IISc Mathematics Initiative, Indian Institute of Science, Bengaluru, Karnataka 560012, India; Centre for Biosystems Science and Engineering, Indian Institute of Science, Bengaluru, Karnataka 560012, India

## Abstract

*Cutibacterium acnes* is a predominant member of the human skin microbiome, generally as a beneficial or a benign inhabitant, which under specific conditions transforms into a pathogen, causing acne and associated inflammatory responses. While there is a wealth of data on the microbiology and genomics of the microbe, there is a lack of comprehensive annotation of the proteins coded by the genome, slowing progress in understanding its unique metabolic versatility and survival in host hypoxic sebaceous niches. We configured an integrated pipeline for functional annotation of the *C. acnes* genome, combining gene calling, domain and ontology mapping, KEGG pathways assignment, proteome-scale structural modeling, predicting pockets and associating the small-molecule metabolites based on binding site similarity to facilitate structure-to-function annotation. Structural predictions using ColabFold covered nearly the full proteome, with 83.7% of models showing high confidence. To infer protein function, ligand-binding sites in the *C. acnes* modeled proteome were predicted using a consensus of three independent algorithms, PocketDepth, SiteHound, and Fpocket. Small molecule ligands were mapped to the resulting pockets through structural comparison of the pockets with known small-molecule binding sites in PDB where there was direct or indirect evidence for such binding, resulting in the *C. acnes* predicted ligandome (CApL) comprising 693 protein-ligand pairs across 534 proteins. CAcnesDB is a comprehensive database of the *C. acnes* genome, hosting functional annotation from multiple levels of input and analyses, covering 2297 protein-coding genes.

## Introduction

*Cutibacterium acnes* is a Gram-positive, facultative anaerobic bacterium that resides near the sebaceous glands inside the pilosebaceous units (PSU) present on the skin [1,2]. It has saprophytic properties and lipolytic activity, owing to which it can acquire nutrients from the secretions of sebaceous follicles, such as sebum lipids. It is a major human skin commensal and produces short-chain fatty acids (SCFAs) such as propionic acid that maintain the skin’s acidic pH and inhibit the growth of pathogenic microbes. The latest classification of *C. acnes* strains includes three subspecies: *C. acnes acnes* (phylotype I, ribotype (RT)- RT1, RT3, RT4, RT5, RT8, RT16, RT532), *C. acnes defendans* (phylotype II, ribotype—RT2, RT6), and *C. acnes elongatum* (phylotype III, ribotype- RT9) [3]. Phylotypes II and III are often found on healthy skin and are non-acneic strains [4]. In contrast, subtypes IA-1 (RT1, RT5, RT532) and IA-2 (RT1, RT4, RT5) are acneic strains and are associated with enhanced virulence, inflammatory potential, and antibiotic resistance [3,5,6]. The first report of the genome sequence annotation of *C. acnes* was in 2004 (Bruggemann et al. 2004; KPA171202 strain 2.56 Mbp, 60% GC content, phylotype IB and ribotype 1) [7]. Since then, about 190 genome sequences of various strains (complete and draft) have been determined, which have indicated the core genome of *C. acnes* that includes both host-beneficial and host-detrimental genes [1,4]. Although this microbe is a part of the healthy skin flora, in an opportune situation, it turns into a pathogen, causing proinflammatory skin diseases, mainly acne vulgaris and implant-associated infections [1]. A comparative genome study between strain 266 of phylotype IA and strain KPA171202 of type IB, identified a number of island-like genomic regions encoding a variety of putative virulence-associated factors, such as dermatan-sulfate adhesin, polyunsaturated fatty acid isomerase, iron acquisition protein HtaA, lipase GehA, as well as genes involved in various transport systems and metabolic pathways [8].

Comprehensive genome annotation is a key requirement for understanding the capability of the organism from a wide range of perspectives, including metabolic adaptation to physiological stimuli, host environmental niches and abiotic stresses, as also for genome comparisons and host-pathogen interactions and biomedical applications, including diagnosis, drug and vaccine discovery. For several organisms, rich annotation using bioinformatics methods has led to the development of databases such as PANTHER, IMG, KEGG, and organism-specific resources such as FlyBase, WormBase, Mycobrowser, TBDB, EcoCyc, and ECMDB, which have greatly aided in collating and organizing information for targeted organisms [9,10]. Although *C. acnes* has been a microbe of interest given how widespread the acne condition is, there is no resource providing comprehensive genome annotation. In the present study, we address this gap and have carried out multimodal multilevel bioinformatics analysis to comprehensively characterize the whole genome and present the results in a dedicated database, CAcnesDB. We anticipate that CAcnesDB will serve as a useful platform for both basic research and translational applications.

## Material and methods

### Sequence analysis, Gene finding, Gene mapping, and Annotation categorization

The whole genome of *C. acnes* KPA171202 was subjected to systematic identification, characterization, and confirmation for each gene candidate. Several tools and algorithms, such as Prokka [11], PGAP [12], Pannzer2 [13], InterProScan5 [14], BLASTp [15], HHblits [16], KEGG tools [17], STRING database [18], VirulentPred [19], P2RP (Predicted Prokaryotic Regulatory Proteins) [20], and DeepTFactor [21] were included in the sequence-to-function assignment protocols (Supplementary Methods Doc1). The full list of tools, algorithms, and databases used for this study is listed in Supplementary Sheet S1. Prokka (Rapid Prokaryotic Genome Annotation) and PGAP (Prokaryotic Genome Annotation Pipeline) were used to generate a first draft of the revised annotation for the whole genome. Both Prokka and PGAP employ the BLAST (Basic Local Alignment Search Tool) algorithm to search against broader databases of proteins, i.e. UniProt [22] and RefSeq [23], respectively. For clarity and mapping consistency, the gene entries in the KEGG database [24] were used as the reference, after mapping gene coordinates from Prokka and PGAP. That resulted in a total of 2369 genes, out of which 55 (6 rRNA + 47 tRNA + 2 tmRNA) were non-coding genes, 17 were pseudogenes, and 2297 genes were protein-coding genes (CDS). All the CDS were further categorized into three subgroups: ‘Hypothetical’, ‘Putative,’ and ‘Known’ proteins based on the search results of the matching words from the existing ‘GenBank’ definition, as illustrated in Fig. S1(a) and a systematic functional annotation was performed (representative example in Fig. S1(b)). The gene grouping was done following the annotation guidelines for prokaryotes, as reported by the NCBI, https://www.ncbi.nlm.nih.gov/genbank/genomesubmit_annotation/. Proteins whose existing GenBank definitions contained the terms ‘putative’ or ‘hypothetical’ were categorized as Hypothetical, whereas proteins annotated as known but containing the term ‘conserved’ were categorized as Putative. The predictions from InterProScan5, with the default thresholds of its member databases, were used to report Pfam and InterProScan domains for the *C. acnes* genome. After the automated genome annotation, results were corroborated with pre-existing GenBank definitions for each gene, where available.

### Genome annotation from structures

Various Software tools, algorithms, and databases used for the ‘annotation from structures’ study, such as ColabFold (MMseqs2 + AlphaFold2) [25], TM-align [26], PocketDepth [27], Fpocket [28], SiteHound [29], SCOP2 [30], and FLAPP [31], are listed in Supplementary Sheet S1. Briefly, the pipeline consists of (a) prediction of protein structure, (b) prediction of pockets, and (c) comparison of binding sites for individual protein sequences in *C. acnes* KPA171202, as shown in Fig. S2.

### Structural models

The genome of the *C. acnes* KPA171202 encodes 2297 protein-coding genes. Structural models were generated using ColabFold, based on the fast homology search of MMseqs2 for generating Multiple Sequence Alignments (MSAs) of the query protein, which is taken as input for the second algorithm, AlphaFold2, to generate predicted structures, from which the best-ranked model is selected for each query protein. The scan ColabFold predicted models for 2280 proteins, nearly the whole proteome (excluding 17, of which 15 proteins have a polypeptide length greater than a thousand residues). Additionally, predicted models for 9 proteins were obtained from AlphaFoldDB, totalling to structural models for 2289 proteins. Structures for the remaining 8 proteins (PPA102, PPA1029, PPA1134, PPA1172, PPA1329, PPA1569, PPA11615, PPA1820) are unavailable in the AlphaFoldDB. Experimentally determined structures were available for 14 proteins which were retrieved from Protein Data Bank (PDB). The list is provided in the Supplementary Sheet S1 (structures PDB-CAcnesDB).

### Quality of the predicted structures

In a modeled protein structure, each amino acid is associated with a per-residue Local Distance Difference Test (pLDDT) score. The pLDDT values for each residue were utilized to assess the quality of the modeled *C. acnes* proteome with the reported thresholds, based on the confidence criteria suggested by AlphaFold developers (highly accurate: regions with pLDDT scores ≥ 90; good confidence: 90 > pLDDT score ≥ 70, low confidence: 70 > pLDDT ≥ 50; uninterpretable: pLDDT < 50), and retained only those regions with pLDDT > 50. Structural alignments were performed using TM-align against (1) modeled structures in AlphaFoldDB, (2) experimentally derived structures in PDB, and (3) non-redundant protein structures present in PDB (m = 26 775). Structurally similar PDB templates with a combined TMScore1 and TMScore2 ≥ 0.5 cutoff were used to assign SCOP2-fold, family, and superfamily information to *C. acnes*’ modeled proteome (n = 2289), as detailed in Supplementary Methods Doc1.

### Pocketome detection

Consensus small molecule binding sites (*C. acnes* pocketome) were predicted using three orthogonal methods: PocketDepth (Geometry, Centrality-based), Fpocket (Voronoi tessellation-based), and SiteHound (Energy-based) [27 − 29]. Putative small-molecule binding sites, which were predicted by all three, were considered to increase the probability of their existence. Predicted pockets in which at least 70% of residues had a pLDDT score ≥ 70 were designated as consensus pockets (CPs) and retained for downstream analysis.

### Binding site comparison

The predicted binding sites (pockets) are aligned against non-redundant PDB binding sites (NrSites) using FLAPP, an in-house fast atomic-level alignment algorithm [31]. Non-redundant PDB binding sites (NrSites) is a set of non-redundant binding sites extracted from the protein-ligand complexes in RCSB-PDB ligands. The protein entries are filtered to 70% sequence identity, and the amino acid residues surrounding the ligand within a 4.5Å distance threshold are extracted, forming the binding site [32]. FLAPP compares two binding sites based on their size and actual residue-residue correspondences, and reports a score (F_min_) and length of alignment (N), where F_min_ ≥ 0.6 and N ≥ 6 indicates significant structural similarity between the sites.

### Ligand association

Our workflow involves a rigorous computational pipeline that includes comparison of predicted pockets with known binding sites in PDB and ligand prediction based on statistically significant binding site similarities, followed by evaluating energetic feasibility of binding of the predicted small molecule. Autodock [33] was run with 10 iterations and the lowest binding energy (free energy change, ΔG) was reported, to assess the binding affinity of a mapped ligand to the predicted pocket of a modeled protein. Protein-ligand pairs having ΔG within the physiological range of -5 to -12 (± 2) kcal/mol [34], were retained to gauge the likelihood of stable binding. To identify the biologically plausible protein-ligand pairs, the *C. acnes* predicted Ligandome (CApL) was derived using experimentally reported ligand-protein associations from literature and chemical fingerprint similarity analysis between predicted ligands and ligands reported for homologous proteins in KEGG [24], ChEMBL [35] and BioLiP [36], computed using OpenBabel [37]. Detailed steps for CApL derivation are described in the Supplementary Methods Doc1.

### Functional annotation

Functional descriptions were assigned by integrating evidence obtained from the sequence- and structure-based annotation analyses. For each protein, results from automated genome annotation, homology and profile-based searches, Gene Ontology, KEGG orthology and pathway mapping, domain analysis, virulence and regulatory predictions, structural classification, and ligand-binding analysis were incorporated. The STRING database was utilized to add the protein–protein interactions, functional annotations, and corresponding KEGG pathways of the *C. acnes* KPA171202 proteome, https://version-12-0.string-db.org/organism/STRG0A28GPG. Based on their functional descriptions, proteins were categorized into nine functional classes: (1) Intermediary metabolism and respiration, (2) Cell wall and cell processes, (3) Information pathways, (4) Conserved hypotheticals and uncharacterized proteins, (5) Regulatory proteins, (6) Detoxification, virulence, and adaptation, (7) Lipid metabolism, (8) Insertion sequences and phages, and (9) Proline-threonine repeats. Iterative manual curation and literature-based up-to-date annotation were carried out to corroborate the assigned annotation across all the evidences for each individual protein. An annotation schema was devised to score the reliability of the assigned functional description (Supplementary Methods Doc1).

### Database and full-stack web development

The CAcnesDB database was developed using Node.js, Express.js, JavaScript, CSS, and HTML to create a data-centric web application (listed in Supplementary Sheet S1). The web interface is developed using Node.js (https://nodejs.org/en). Node.js [38] is a server-side JavaScript runtime environment that builds scalable and efficient applications that interact with databases and handle data operations. Express.js [39] is a web application framework for Node.js that simplifies the process of building robust and modular web applications. JavaScript was used for both server-side (Node.js) and user-side (browser) scripting. The web application used CSS (Cascading Style Sheets) to style visual aspects such as layout, colors, and typography. HTML (Hypertext Markup Language) was used to structure and present the contents on the web. The combination of these technologies was utilized to create a Full-Stack Web Development, which facilitates a seamless user experience, managing and storing data locally in JSON files as well. Additionally, STRING API [18] was integrated with the CAcnesDB, which provides an interactive display for protein-protein network visualization. Mol*(https://molstar.org/) [40] was used to represent the protein structures in 3 dimensions. It is a collection of tools that have been designed to visualize and analyze molecular structures in 3D space. It provides interactive and dynamic representations of the molecular structures. The database can be queried using KEGG IDs, gene symbols, UniProt IDs, NCBI IDs and CAcnesDB functional annotations.

Database URL: https://proline.biochem.iisc.ac.in/CAcnesDB/

## Results

### Overview of the genome annotation

We configured a computational pipeline for genome annotation, comprising sequence analysis of the gene products, protein structure analysis, binding site structural analysis, 3D pocket-based ligand identification, and database development (Fig. 1). The assignment of functional descriptions to each protein was made systematically, based on the results obtained from individual analyses. The annotations were manually cross-checked with the existing ‘GenBank’ definitions obtained from the entries in KEGG, and updated with new information from the various steps comprising the protein characterizations based on sequences and modeled structures. Next, we consolidated the evidence for functional annotation from multiple levels of analyses, to get updated genome characterization and protein annotation at high resolution and present them in a database, CAcnesDB. We indicate the confidence from the multiple evidences for the functional descriptions, using an annotation scoring schema.

**Figure 1:**
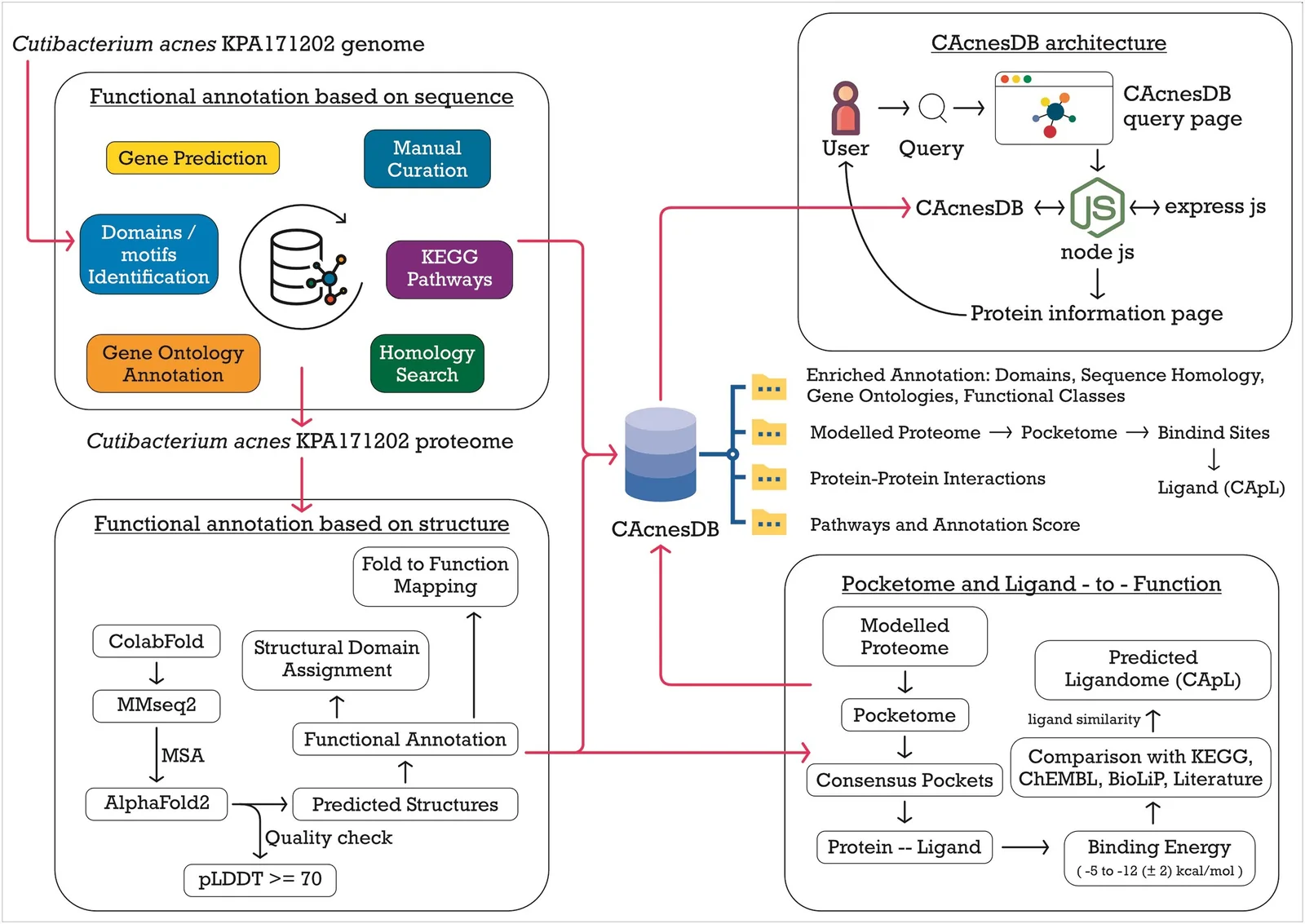
A schema representing an integrated pipeline comprising tools and databases utilized for genome annotation from sequences and structures, and construction of the CAcnesDB. Different aspects of annotation are illustrated in the schema.

### Summary statistics

Of the 2297 protein coding genes in the genome, domains were assigned for 1958, sequence-sequence homology mapping was obtained for 1721, profile-profile sequence matches obtained for 2297 and structural models were predicted for 2289, binding sites predicted for 2205, ligands predicted for 1865, protein-protein interactions predicted for 1890 proteins, put together providing functional annotation for 2072 proteins (Detailed statistics in Supplementary Sheet S1- summary stats). Overall, the CAcnesDB database contains annotations for the *C. acnes* KPA171202 strain, associated KEGG orthologues, KEGG pathways, GO terms (BP, MF, and CC), SCOP2 hierarchies, functional class, and evidence-based annotation scores. A comparison of the results in CAcnesDB with the corresponding data from public repositories is provided in Table 1. The general statistics of the results in CAcnesDB are listed in Supplementary Table S1 and sequence-based annotations are in Supplementary Results Doc2.

**Table 1:** Summary statistics of the availability of results from each analysis carried out in the annotation of the C. acnes KPA171202.

	KEGG	^b^UniProt	^b^NCBI & TBdb	^a^CAcnesDB
**Protein coding genes (CDS)**	2297	2295 (299 reviewed + (1996 unreviewed)	2425, 2297	2297
**Pseudogenes**	17	–	88, -	17
**Protein non-coding genes**	54 (9 rRNA + 45 tRNA)	–	76 (18 riboswitch, 1 RNAse_P_RNA, 9 rRNA, 1 SRP_RNA, 2 tmRNA, 45 tRNA), -	55 (6 rRNA + 47 tRNA + 2 tmRNA)
**Protein with ‘probable/ putative/ predicted’ functional assignments**	472 (468 ‘putative’+16 ‘probable’+12 ‘predicted’)-(24 ‘hypothetical’)	114	3, 441 (444 ‘putative’+ 10 ‘probable’+11 ‘predicted’)-24 ‘hypothetical’)	215 (194 ‘putative’ + 14 ‘probable’+ 7 ‘predicted’)
**Hypothetical/ uncharacterized/domain of unknown (DUF) proteins**	481	357	425, 648	256 (225 are newly annotated)
**Protein with gene symbols**	–	442	- , 58	823
**Protein with assigned domains**	2085 (Pfam)	1981 (Pfam, InterPro)	- , 1841 (Pfam, Tigrfam)	2085 (InterPro, Pfam, Gene3D, CDD, Coils, HAMAP, PRINTS, PrositePatterns, PrositeProfiles, SFLD, SMART, superfamily, Tigrfam, MobiDB, Panther)
**Protein with assigned gene ontology (GO) (BP or MF or CC)**	–	1759	1267, -	1686
**Protein with assigned KEGG orthology**	1397	–	–	1503
**Protein with assigned KEGG pathways/modules**	909 (KEGGpathways+KEGG modules)	–	–	1596 (KEGG pathways+KEGG modules)
**Structural models for proteins**	–	2286 (AlphafoldDB)	–	2289
**SCOP domains**	–	–	–	1208 (Superfamily and/or FOLD and/or Family)
**Availability of ligand-to-function annotation**				Yes
**Availability of functional annotation for the whole genome of *C. acnes***	–	–	–	Yes
**Functional classes**	–	–	–	9 Functional Classes
**Availability of annotation-confidence schema**	–	Yes	–	Yes

a Manually curated, known non-redundant dataset.

b Information retrieved in original form without redundancy filtering.

*Notes*: The results are compared with the available data from the other public resources, i.e. KEGG, UniProt, NCBI, and TBDB.

### Sequence-based annotations

The sequence-based function annotation for a gene comprised information from one or more of the following: (a) Gene finding: functional annotation obtained from the Prokka and PGAP analyses, (b) Homology search: functional description of the top-hits obtained sequence-sequence and profile-profile alignments, (c) Gene Ontology (GO): obtaining functional role from the combined knowledge of Biological Processes (BP), Molecular Function (MF), and Cellular component (CC), (d) Pathway analysis: deriving the functional role from KEGG orthology (KO) and pathway information, (e) Domains: Establishing the functional capacity from the combined domains associated with a protein sequence, (f) Virulence: identifying the functional capacity of a protein for its virulence or regulatory activity, and (g) Miscellaneous: Literature-based annotation derivation for other available protein functional terms.

Sequence-based homology identified functions for 1 721 CDS, while profile-profile searches yielded functional descriptions for 2 297 genes. Many proteins are made of one or more domains, whose functional annotations are available from Pfam and InterProScan databases. Since sequence matches become more sensitive at the domain level, we have systematically identified domains and mapped them to Pfam and InterProScan. Domains were identified for a major portion of the *C. acnes* genome. Out of 2297 protein-coding genes (CDS), 1872 (≈ 81.5% coverage) were associated with Pfam domain IDs, and 938 (≈ 40.8% coverage) were associated with CDD domain IDs. The overall output from the InterProScan is listed in Supplementary Sheet S1 (Summary stats).

Fig. S3(a) summarizes the distribution of three GO terms across the whole genome in *C. acnes* KPA171202. In total, 149 genes could be associated with all three GO terms. Fig. S3(b) displays the distribution of GO (BP) categories among the 1 036 annotated predicted protein-coding genes. The most abundant GO: BP were transmembrane transport, carbohydrate metabolic process, and cellular amino acid metabolic process. Fig. S3(c) displays the distribution of GO (MF) categories for 1 566 annotated predicted protein-coding genes. The top GO: MF terms were transferase activity, hydrolase activity, and oxidoreductase activity. Fig. S3(d) displays the distribution of GO (CC) categories for 326 protein-coding genes. The most abundant GO: CC were the plasma membrane and ribosome, followed by the organelle and the extracellular region.

Protein-protein interactions were obtained from STRING-db [18] for the *C. acnes* KPA171202 proteome, from which genes were mapped to various KEGG pathways. KEGG orthology (KO) prediction resulted in the association of KO for 1503 CDS, and STRING-db associated KEGG pathways for 1593 proteins. Integration of KEGG pathway and module annotations resulted in associations with 1596 proteins. Next, using P2RP (Predicted Prokaryotic Regulatory Proteins tool) [20], 12 histidine kinases and 12 response regulators were identified. Further using DeepTFactor, which employs three parallel convolutional neural networks, 106 proteins were predicted to be transcription factors. Using VirulentPred [19], 1622 proteins were associated with virulence, (Supplementary Sheet S1 (Summary stats)) and an overlapping set of 988 proteins were identified from the Virulent Factor Database (VFDB).

### Enriching genome annotation with 3D structures

Next, in order to utilise structural information in *C. acnes* genome annotation, structural models were predicted at a proteome scale, using ColabFold and AlphaFold2 (Supplementary Sheet S1, (Fig. S4), which resulted in models for 2289 proteins. ColabFold and AlphaFoldDB both rely on AlphaFold2, but ColabFold generates structures using a standardized MMseqs2-based MSA pipeline, leading to a more uniform sequence-search framework. A comparison of ColabFold and AlphaFoldDB models showed high overall concordance, with 83% and 67% of structures differing by < 3 Å and < 2 Å RMSD, respectively; cases with larger deviations were largely attributable to alternative inter-domain orientations while conserving domain-level structures, with minimal impact on functional annotation. The quality of the ColabFold-predicted structures for the *C. acnes* proteome was assessed using the predicted local distance difference test (pLDDT), a residue-level confidence metric reported by AlphaFold2. Of the 2289 protein structures, 1918 (83.7%) had at least 70% of their residues with pLDDT scores > 70, indicating that the majority of the proteome had substantial high-confidence structural coverage (Supplementary Sheet S2). At the residue level, 67.4% of residues were predicted with high confidence, 18.9% with intermediate confidence, 5.9% with low confidence, and 7.8% had pLDDT values below 50, indicating unreliable structural predictions (Fig. S5).

The top-ranked predicted structures for each of the 2289 proteins constituted the *C. acnes*- structural proteome and were used for the prediction of the pocketome and associated ligands. Structural folds were assigned to the 2289 models by using TM-align (Supplementary Sheet S1). The most abundant folds identified in the modeled proteome included TIM β/α barrel, Rossmann-like, ferredoxin-like, OB-fold, and UBA-type three-helical bundle architectures (Fig. S6).

### *C. acnes* pocketome

To predict possible small-molecule ligand binding pockets, three binding pocket prediction methods were used: Depth-based (i.e. PocketDepth), Energy-based (i.e. SiteHound), and Voronoi tessellation-based (i.e. Fpocket). All three methods were run independently on each of the 2289 modelled structures (3 × 2289 = 6867). The numbers of pockets predicted by each of the methods are PocketDepth: 52 555, SiteHound: 22 890, and Fpocket: 26 483. From these, consensus pockets (CP) were identified as described earlier (Fig. S2). The CPs from the Fpocket output are used for this analysis, and only those Fpocket pockets with at least 4 residues that match the pockets predicted by PocketDepth and SiteHound were considered further. Based on the pLDDT scores, the percentage of high and intermediate confidence residues (pLDDT ≥ 70) with respect to the total number of residues in the pocket was calculated to evaluate the quality of the individual pockets. Among all the pockets, about 77.58% are located in protein structure regions with relatively high and intermediate confidence (ratio ≥ 0.7). This resulted in 11 339 CPs spanning over 2044 proteins, which together constitute the *C. acnes*’ Pocketome.

### C. *acnes* predicted Ligandome, CApL

The Consensus Pockets (CPs) thus obtained were then compared to the known binding sites (NrSites) derived from the Protein Data Bank (PDB), using FLAPP, with a stringent cutoff of F_min_ ≥ 0.6 and number of alignment ≥ 6. Based on the analysis, the obtained CPs were recognized to have the binding capacity for the corresponding ligands associated with the structurally aligned PDB templates. Binding energies (ΔG) for all ligand–protein pairs were calculated using AutoDock. A total of 4 738 protein–ligand pairs were obtained with ΔG values within the physiological range (−5 to − 12 ± 2 kcal/mol), comprising 1 794 unique proteins. Further, to reduce the risk of overinterpretation, we derived biologically plausible protein-ligand pairs, having experimental evidence for related proteins (reported in literature and/or matched with the ligand annotations documented in BioLiP, ChEMBL, KEGG databases), which forms the final list of CApL. Matching the predicted *C. acnes* protein-ligand pairs against ChEMBL, KEGG, BioLiP and literature identified 65, 403, 456, and 232 pairs, respectively, yielding 693 unique biologically plausible protein-ligand pairs. This set, defined as the *C. acnes* predicted ligandome (CApL), comprising 534 proteins and 221 ligands (Fig. 2A). As the analysis has been conducted at the genome scale, the predicted ligands that bind across the consensus pockets of the *C. acnes* pocketome, qualitatively comprise the predicted Ligandome in *C. acnes* (CApL).

**Figure 2:**
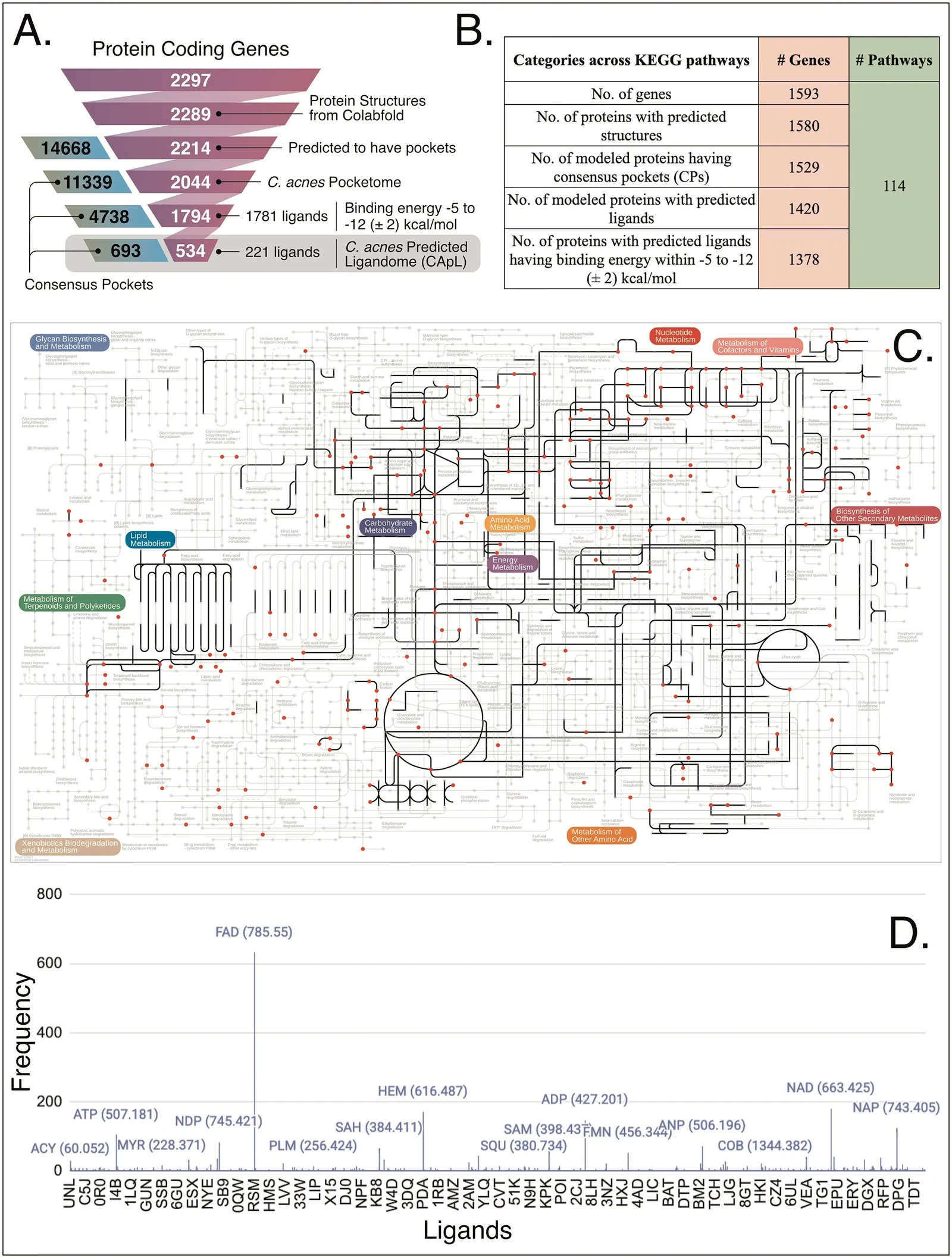
(A). The computational pipeline used for Proteome, Pocketome, and predicted ligandome identification for the *C. acnes* interactome profile. The pipeline involved stepwise filters based on the modeled proteome, Consensus Pockets (CP) identification, and CApL analysis. (B) A Table showing the coverage of genes, modeled protein structures, CPs and the confident ligand associations from CApL, available across the KEGG pathways. (C) Metabolic map of central metabolism in *C. acnes*, indicating extensive coverage of ligand annotation in the *C. acnes* predicted ligandome. The edges colored in black indicate the availability of protein structure catalyzing the reaction, and the nodes colored in red represent the small ligand molecules taking part in the reaction for which the binding site has been mapped onto the respective protein structure. The Fig. is generated using ipath3 [55]. (D) An illustration of ligand associations for *C. acnes* Confidence Pocketome. The frequency on the Y-axis indicates the number of occurrences of the binding site of that ligand in the *C. acnes* pocketome.

A subset of the CApL protein–ligand pairs associated with metabolic pathways was specified as the predicted metabolome. The results for ligand similarity analyses between the predicted and reported pairs in KEGG, BioLiP, ChEMBL, literature, and derivation of CApL are provided in Supplementary Sheets S2 and S3. The coverage of the structural models and their associated ligands is represented in terms of KEGG pathways in Fig. 2B. The protein-ligand associations from our analysis are mapped onto the *C. acnes* KEGG metabolic map (Fig. 2C), which illustrates substantial coverage of the cellular metabolic network. The ligands are projected onto a plot based on their molecular weight, reflecting a predicted mass-spectrometry-like metabolome spectrum (Fig. 2D). The most frequently observed ligands are FAD, followed by NAD, ADP, HEM, NAP, and ATP. The predicted ligands that can bind to the *C. acnes* pocketome span a wide range of molecular weights, the lightest detected being acetate (ACY, 60.052) to one of the heaviest being Co-methylcobalamin (COB, 1344.382).

### Annotation quality

Annotations present the challenge of describing the annotation quality. This was dealt with by designing a scoring method to guide the annotation by identifying pieces of information and whether the assigned annotation is valid. Keeping this in mind, an annotation score was developed, where a value of ‘1’ was given if a particular piece of evidence was found to be making a connection with the given functional description; a score of ‘0’ was given for the lack of any such evidence, as outlined in Fig. 3A. An example of computation of annotation-score for the assigned annotation in PPA0075 is shown in Fig. 3B. Summarization of the annotation score across the three categories showed 361 genes from the ‘Known’ category, which received an annotation score of ‘7’ (Fig. 3C). This shows evidences from all seven categories (‘correlation with the existing annotation’, ‘automated annotation’, ‘homology searches’, ‘KEGG orthology’, ‘Gene Ontology’, ‘Domain-based correlation’, and ‘Ligand to function’) correlated with the assigned functional descriptions. The genes from the ‘Hypothetical’ category received a ‘0’ annotation score, showing the absence or no correlation of any evidence with these genes. The largest fraction, 600 genes from the ‘known’ category, received an annotation score of ‘6’, which underlines the rigorous steps followed in our annotation pipeline. The complete list of annotation scores for all proteins with their individual evidence categories is given in Supplementary Sheet S4 (annotation scores).

**Figure 3:**
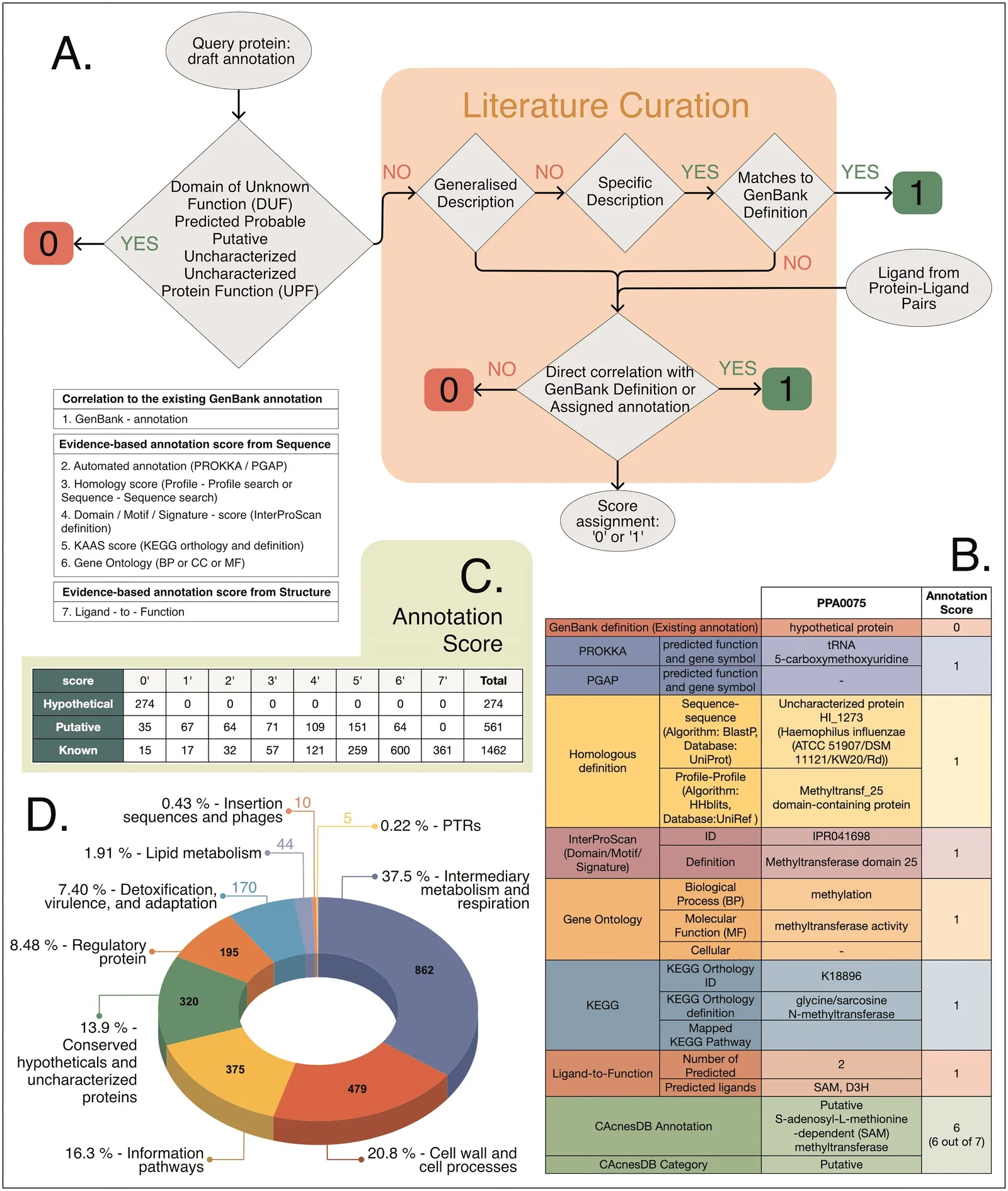
(A) Evidence-based annotation scoring schema. The figure illustrates the assignment of the annotation score to the revised/assigned annotation based on seven sections of the annotation pipeline. The steps were followed for each of the individual proteins in the proteome. Each step shows the assignment of either score ‘1’ or ‘0’ based on the match (presence/absence) to the particular section. Assessment of the annotation score was conducted with literature-based verification, wherever necessary. (B) Annotation-score assignment shown with an example of PPA0075; where it was annotated to be a ‘Putative SAM-dependent methyltransferase’ and was given a score of 6. (C) Distribution of annotation score across the three categories: Hypothetical, Putative, and Known. (D) Functional classification of *C. acnes* KPA171202 genes.

### Consolidated annotations

The database, constructed specifically for *C. acnes* research, presents the consolidated annotation available for each protein. The protein-encoding genes were classified into nine functional classes (Fig. 3D). These are (1) Intermediary metabolism and respiration, (2) Cell wall and cell processes, (3) Information pathways, (4) Conserved hypotheticals and uncharacterized proteins, (5) Regulatory proteins, (6) Detoxification, virulence, and adaptation, (7) Lipid metabolism, (8) insertion sequences and phages, and (9) proline-threonine repeats. Some proteins belong to more than one functional class. The largest functional category was observed to be ‘Intermediary metabolism and respiration’, containing 862 enzymes, 766 of which were known and 96 were newly added/improved from our annotation.

Some examples where annotation is enriched from our work are (i) PPA0083 is identified as ‘Putative 1,3-beta-galactosyl-N-acetyl hexosamine phosphorylase’ (as compared to the label of ‘hypothetical protein’ in the previous annotation), (ii) PPA2025 is ‘Putative FMN reductase (NADH) RutF’ (as compared to ‘hypothetical protein’ in the previous annotation), (iii) PPA1688 is ‘Putative phosphoheptose isomerase’ (as compared to hypothetical protein in the previous annotation). The different aspects of annotation for all the given examples are listed in Supplementary Sheet S4.

The ‘Cell wall and Cell Processes’, was another major functional category, that has all the ABC transporters, membrane permeases, and proteins facilitating cell divisions. This class includes 410 known and 69 new/improved annotations. Examples include (i) PPA0637 encoding ‘Putative DivIVA domain-containing protein’ as compared to ‘hypothetical protein’ in the previous annotation, (ii) PPA0231 is ‘Putative pilus assembly protein TadB’, as compared to hypothetical protein in the previous annotation). Another key functional category is ‘Information Pathways’, which includes genes essential for maintaining genetic information and supporting core cellular functions. This category encompasses genes involved in DNA modification and repair, as well as RNA subunits that play roles in replication, transcription, and translation.

Regulatory proteins, such as transcription factors, histidine kinases, and response regulators, play a central role in controlling the signaling pathways that enable the bacterium to adapt to environmental stress and regulate the expression of virulence factors. Genes involved in these adaptive responses, including those related to detoxification and virulence, are categorized under the functional class of ‘Detoxification, virulence, and adaptation’.

The smallest portion of the genome, i.e. the PTR class, has five genes, which have been shown to be involved in colonization/adhesion/inflammation [41, 42, 43, 4,4]. PPA2127 has PT repeats at its C-terminus and was found to be Dermatan sulfate-binding adhesin 1 (DsA1), which is a host cell-surface attachment protein having dermatan-sulphate-binding activity and immunoreactive properties. PPA2210 and PPA2270 encode Dermatan sulfate adhesin (DsA2) and L, D-transpeptidase LppS- a putative secreted lipoprotein, respectively. PPA1715 was identified as TQXA, a thioester domain-containing protein [42]. Dermatan sulfate/chondroitin sulfate (DS/CS) are skin glycosaminoglycans that mediate the adherence of bacteria to epithelial cells, including staphylococci, and participate in infections and mediate inflammatory response [44]. Transposases (PPA0858, PPA0876, PPA1974, PPA2354, PPA2355, PPA2377, PPA2391) and conserved phage-associated proteins (PPA1069, PPA1594, PPA1604) are placed under the class for ‘insertion sequences and phages’.

Put together, our work has resulted in enriched annotations for 2072 proteins (representative annotations are illustrated in Table 2 and Fig. 4), of which 225 proteins that were previously identified as hypothetical proteins, are assigned new functional annotations. Some of these newly annotated proteins belong to ‘Regulatory proteins’, examples being (a) PPA2204 encoding for ‘Putative transcriptional regulatory, WhiB’, (b) PPA0329 encoding for ‘Putative transcriptional regulator (RNA polymerase sigma factor)’.

**Figure 4:**
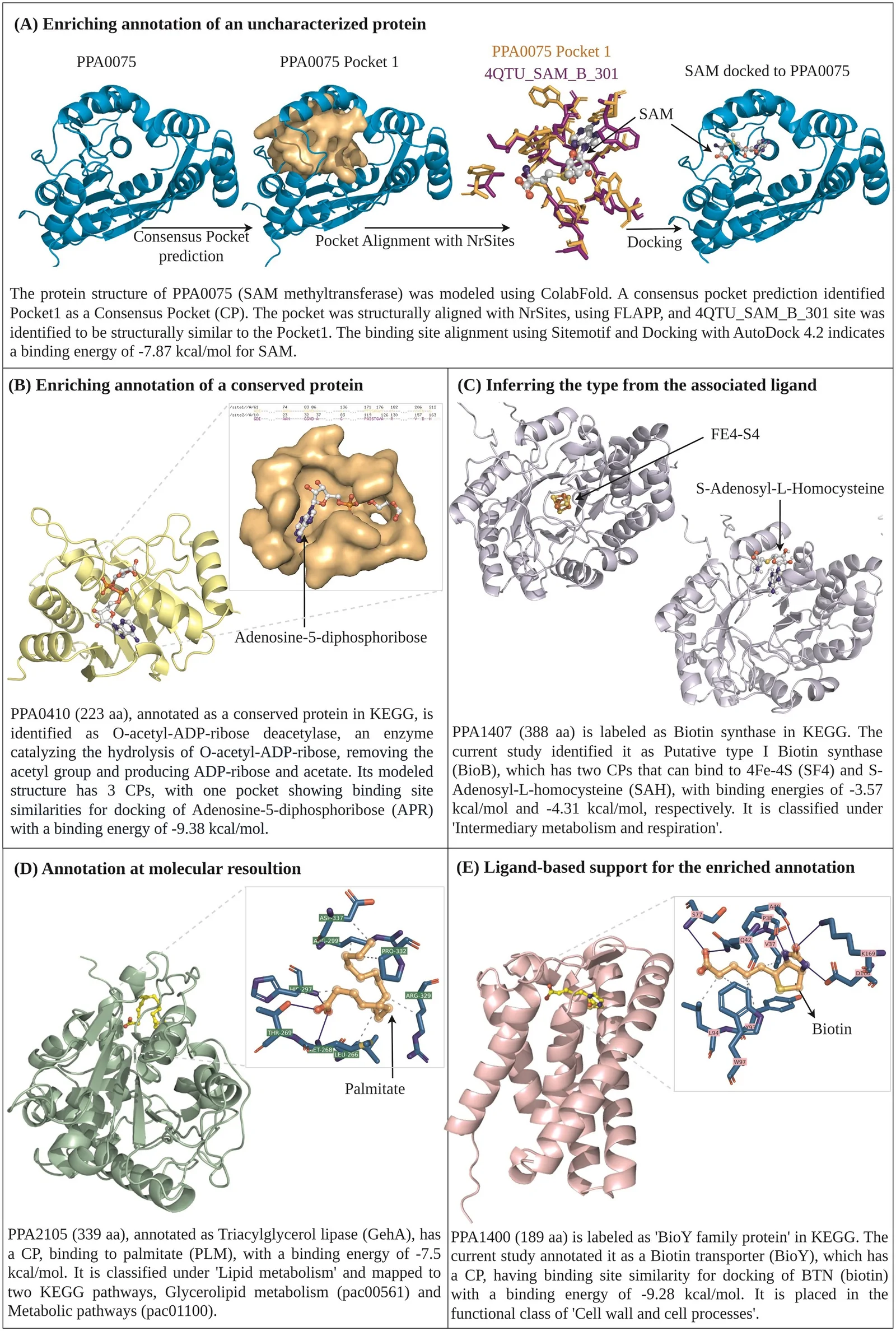
Different aspects of annotations are shown in the Table, (A) from assigning the putative function to an uncharacterized protein (PPA0075), (B) to assigning a annotation for a conserved proteins (PPA0410), (C) inferring the type of enzyme from the associated ligand (PPA1407), (D) finding the substrate for the enzyme catalysis (PPA2105) and (E) confirming the existing annotation with the associated ligand (PPA1400).

**Table 2:** A representative example of consolidated annotation derived from sequence- and structure-based analyses

**PPA0018 Previous annotation:** (GenBank) sugar kinase **Current annotation:** Ribokinase (RbsK)
**Sequence analysis:** Sequence-sequence homology analysis for PPA0018 identifies Ribokinase (RbsK) from Schizosaccharomyces pombe (UniProt: O60116) as the closest hit, with 37.2% sequence identity over 309 aa, with 95% query coverage and a highly significant E-value of 2.16 × 10⁻⁴⁶. Prokka and PGAP also annotate PPA0018 as Ribokinase (RbsK). Profile-profile homology search using HHblits against UniRef100 further supported its annotation as a ribokinase. Domain analysis identified the PfkB family carbohydrate kinase domain (PF00294), with InterProScan identifying IPR011611 (Carbohydrate kinase PfkB) along with IPR029056, IPR002173, IPR002139 and IPR011877.
**Structure analysis:** PPA0018 is 309 aa long; the predicted structural model covers the complete polypeptide chain, with 302 residues (97.73%) having pLDDT scores ≥ 70, indicating high confidence. SCOP2 classification assigned PPA0018 to the YjeF C-terminal domain-like fold, superfamily, and family.**Pocket analysis** identified putative binding sites for α-D-ribofuranose (RIB), GTP/ATP. The RIB pocket involves residues ASN15, ASP17, GLY43, LYS44, ASN47, ALA97, GLU142, GLY251 and ASP254, with a predicted binding energy of -3.99 kcal/mol, whereas the GTP/ATP-binding pocket involves ALA187, LEU188, LEU191, SER198, ARG199, LEU200, and GLU201, residues with a predicted binding energy of -3.38 kcal/mol.
**Functional analysis:** Gene Ontology annotations associated PPA0018 with the biological processes carbohydrate phosphorylation (GO:0046835) and D-ribose catabolic process (GO:0019303), and with the molecular functions ATP binding (GO:0005524), ribokinase activity (GO:0004747) and metal-ion binding (GO:0046872). Cellular component annotation placed the protein in the cytoplasm (GO:0005737). KEGG assigned PPA0018 to K00852, corresponding to dehydrogluconokinase/ribokinase, and associated it with the pentose phosphate pathway (pac00030) and metabolic pathways (pac01100).**Virulence:** Predicted as virulent by VFDB and non-virulent by VirulentPred.**Regulatory role:** No regulatory role, based on P2RP and DeepTFactor.**Reaction:** Ribokinase activity corresponding to K00852 [EC:2.7.1.15], catalyzing the phosphorylation of D-ribose to D-ribose-5-phosphate using ATP as the phosphoryl donor, producing ADP.Functions from network analysis: STRING analysis identified associations with RbsD (combined score: 0.954), PPA0017 (0.926), Prs (0.926), and transketolase (0.920), where the combined score represents the overall confidence of a functional or physical association between the protein pairs.
**Consolidated annotation:** PPA0018 is annotated as a ribokinase (RbsK), with integrated sequence, domain, functional, structural, and network analyses supporting its role in ribose metabolism and the pentose phosphate pathway. This gene is assigned to the ‘Intermediary metabolism and Respiration’ functional class.

### CAcnesDB database functionalities

The CAcnesDB home page provides access to various aspects of annotation, namely (i) Protein Details, (ii) Domain and Gene Ontology, (iii) Protein Structure and Binding Pockets, predicted ligands and the corresponding binding energies (iv) Protein-Protein Interaction (v) Pathway Information. The database is searchable in multiple ways, through the listed gene symbols, KEGG IDs, UniProt IDs, NCBI IDs, or assigned annotations. CAcnesDB presents modeled protein structures (ColabFold predicted top-scoring modeled structure for each query protein) for 2 289 protein sequences that can be assessed in the ‘Protein Structure and Binding Pockets’ tab. In addition, the tab provides the list of predicted CPs for the queried protein. The ‘downloads’ tab provides assessed, biologically plausible protein-ligand pairs that can be visualized with an interactive graphics interface (Fig. S7).

## Discussion

Genome annotation is an iterative activity and periodic updation is necessary to benefit from the knowledge that is continuously being accumulated in databases and primary literature for many organisms. A number of draft genome sequences of *C. acnes* strains have been reported, indicating the interest in studying this organism, and we expect our annotation reported here to be widely useful to *C. acnes* researchers. The annotation pipeline devised by us uses a combination of automated and manually curated updates of the whole genome characterization. The enriched annotations involve various aspects of gene characterization, which were extracted from both the sequence and structure-based analyses. While the sequence-based methods provided functional descriptions in terms of Gene Ontology (BP, MF and CC) classes, KEGG pathways, virulence and regulatory role predictions, the structure-based methods provided a deeper layer of evidence, by identifying possible ligands associated with each pocket, defined with the exact amino acid residues lining them, at a proteome scale.

A total of 25 experimentally determined crystal or solution structures of *C. acnes* proteins were retrieved from the PDB [45–51], of which 23 entries mapped to 14 proteins in *C. acnes* KPA171202. Enhancements to the annotation for these proteins are listed in Supplementary Sheet S1. For example, PPA1560 was annotated as a surface-associated sialidase based on structural, functional, and ligand-binding evidence. Experimentally determined structures available in the PDB include the ligand-free form (PDB: 7LBU) and a transition-state complex bound to Neu5Ac2en (PDB: 7LBV) [51]. Consistent with this annotation, CAcnesDB identified a putative binding pocket accommodating DAN (2-deoxy-2,3-dehydro-N-acetylneuraminic acid) with a predicted binding energy of − 5.04 kcal/mol. Domain analyses further supported this assignment through the identification of BNR repeat-like domains (PF13088), conserved sialidase-associated signatures (IPR036278, IPR026856, IPR011040), and the exo-α-sialidase activity term (GO:0 004 308). Structural classification based on SCOP fold and family information revealed the presence of immunoglobulin-like β-sandwich, galactose-binding domain-like, and six- and seven-bladed β-propeller architectures, which are characteristic features of sialidases [52]. Furthermore, PPA1560 was classified under ‘intermediary metabolism and respiration’ with a high confidence score of 7 in the ‘known’ functional category, collectively supporting a robust functional annotation for this protein.

Several ABC transporters (pac02010) and membrane permeases that were placed under the functional class of ‘cell wall and cell processes’ were found to have pockets that can bind to various skin-related metabolites, such as squalene (SQL), sebum lipids like oleic acid (OLA), myristic acid (MYR), and vitamins. The knowledge of the specific substrates that can bind to the transmembrane transporters and permeases serves as hypotheses to study metabolite-centric host-pathogen interactions between *C. acnes* and human skin. One of the characteristic features of *C. acnes* is having PT repeats (PTRs) that possibly have immunogenic properties [41,42], similar to the PPE repeats in *Mycobacterium tuberculosis* [53]. Previous reports (McLaughlin et al., [4]) have indicated PPA2210, PPA1715, PPA1879, PPA1880, PPA1881, PPA1906, PPA2130, and PPA2270 to contain PTRs. From our annotation, we have grouped five proteins in the PTR class, namely, PPA2127, PPA2210, PPA1880, PPA1715, and PPA2270. These genes encode adhesins (DsA1, DsA2), a secreted lipoprotein, and have also been placed under the class of ‘Detoxification, virulence, and adaptation’, due to their involvement in inflammatory responses and infections. Given that *C. acnes* phages exhibit a narrow host range, including strains such as *C. acnes* KPA171202 [54], conserved phage-associated proteins and transposases were classified under the category of ‘insertion sequences and phages’.

In conclusion, the genome annotation of *C. acnes* has been revisited using a multi-analysis pipeline, resulting in annotation for 2297 proteins with significant improvement in 2072 proteins. The multi-modal approach employed in the current study, including pathway mapping, domain and fold identification, and ligand prediction, provides a robust foundation for annotating the previously uncharacterized proteins and adding higher resolution information to the proteins where some annotation was available. The analyses and the annotations are presented in CAcnesDB (https://proline.biochem.iisc.ac.in/CAcnesDB/), an online database providing integrated access to genome sequence, structural proteome, and literature curation for *C. acnes*. CAcnesDB stores hierarchical functional and structural characterization, i.e. modeled proteome, protein-protein interaction network, pocketome, and ligandome. CAcnesDB provides a suite of tools for searching, browsing, analyzing, and downloading the data. CAcnesDB also serves as a platform to add future genome annotations of related strains and perform a comprehensive analysis. By integrating a wide range of genomic data with tools for their use, CAcnesDB is a unique platform for both basic science research in *C. acnes*-related skin infections and disorders, as well as research into the discovery and development of drugs and possibly vaccines for acne vulgaris.

## Supplementary Material

baag067_Supplemental_Files

## Data Availability

CAcnesDB is accessible at https://proline.biochem.iisc.ac.in/CAcnesDB/ and on GitHub https://github.com/nchandra-iisc/CAcnesDB.
