# Supplementary material for: CAcnesDB: a database of Cutibacterium acnes with integrated functional insights derived from multi-modal genome annotation
Source: Database (Oxford). 2026 Nov 10;2026:baag067. doi: 10.1093/database/baag067 (PMC13750175; doi:10.1093/database/baag067)
Supplement: Supplementary file 1 [file baag067_supplemental_files.zip › Supplementary Results Doc2.docx]

# **Supplementary Results**

## Annotation Overview and Summary Statistics

|  | **CAcnesDB** |
| --- | --- |
| **# Protein coding genes**  **(CDS)** | 2297 |
| **# Pseudogenes** | 17 |
| **# Protein non-coding genes** | 55 (6 rRNA + 47 tRNA + 2 tmRNA) |
| **# Protein with definite functional assignments**  **(absence of ' Putative/ Probable/ Predicted/ Hypothetical')** | 1826 |
| **# Protein with 'Probable/ Putative/ Predicted' functional assignments** | 215 (194 'putative' + 14 'probable'+ 7 'predicted) |
| **# Hypothetical/ Uncharacterized/ Domain of Unknown (DUF) proteins** | 256 (225 are newly annotated) |
| **# Protein with gene symbols** | 823 |
| **# Protein with assigned domains** | 2085 (InterPro, Pfam, Gene3D, CDD, Coils, HAMAP,  PRINTS, PrositePatterns, PrositeProfiles, SFLD, SMART,  superfamily, Tigrfam, MobiDB, Panther) |
| **# Protein with assigned Gene Ontology (GO) (BP or MF or CC)** | 1686 (BP: 1036, MF: 1566, CC: 326) |
| **# Protein with assigned KEGG orthology (KO)** | 1503 |
| **# Protein with assigned KEGG pathways/modules** | 1596 (KEGG pathways + KEGG modules) |
| **# Structural models for proteins** | 2289 |
| **# Protein with SCOP domains** | 1208 (Superfamily and/or FOLD and/or Family) |
| **Availabilitiy of Ligand-to-Function Annotation** | Yes |
| **# Protein-ligand pairs within intermolecular binding energy, -5 to -12 (± 2) kcal/mol** | 4738 |
| **Availability of Functional for the whole genome of C. acnes** | Yes |
| **# Functional Classes** | 9 Functional Classes |
| **# Proteins with Functional Classes** | 2273 |
| **Availability of Annotation-confidence schema** | Yes |

**Table S1**: Summary statistics of the availability of results from each analysis carried out in the annotation of the *C. acnes* KPA171202.

**Figure S3**: Pie chart representing the Gene Ontology (GO) analysis from Pannzer2 (a) Association of GO terms for *C. acnes* KPA171202 genome in three principal GO categories (BP: Biological Process, MF: Molecular Function, CC: Cellular Component). (b) The distribution of GO terms for the BP category across 1036 genes. (c)The distribution of GO terms in the MF category for 1566 genes. (d) The distribution of GO terms in the CC category for 326 genes.

## Structure-based Analysis

**Figure S4:** Comparison between the AlphaFoldDB and ColabFold modeled structures. A) The distribution of RMSD values across the number of ColabFold models. The cumulative distribution shows that 83 % < 3Å and 67% < 2Å, over their entire polypeptide chains. Superposition of the modeled structures from AlphaFoldDB (blue) and ColabFold (red) for example proteins, B) PPA1627 and C) PPA2186, with RMSDs < 1Å. For structures with RMSD > 3Å, the domains are largely superposable with slightly different orientations, as shown in the example D) PPA1316.

**Modeled proteome’s quality assessment**

**Figure S5:** Coverage of the proteome by different ColabFold pLDDT score thresholds.

**Summary of the associated FOLD, superfamily, and family**

Unlike traditional homology modeling methods, AlphaFold does not give other information, such as the template structure used, making it difficult to assign functional hierarchies, such as from the SCOP database. Further, template structures are usually annotated and help transfer functions to the modeled protein structure. A structural alignment exercise was performed to assign fold information to *C. acnes* (n=2289), where 2289 proteins were aligned against non-redundant protein structures present in PDB. The program TMalign was used to establish the scan. This resulted in a 6,12,87,921 combination of pairs, which is roughly equivalent to 61 million pairs of structural alignments (see [Sheet S1 (summary stats)](https://docs.google.com/spreadsheets/d/1z6P2PB2_h9N4WwovIBCHk1OZ1659b_6xj9P7S8GcCGU/edit?gid=0#gid=0)). The top folds (Figure S5(a)) in the modeled *C. acnes* proteome include TIM beta/alpha-barrel, Rossmann(2×3)oid, Ferredoxin-like, OB-fold, UBA-type 3-helical bundle, etc. The top superfamilies (Figure S5(b)) in the modeled proteome include ‘Aldolase’, ‘Dihydropteroate synthetase-like’, and ‘Phosphoenolpyruvate/pyruvate domain’.

(a) SCOP2: FOLD

(b) SCOP2: Superfamily

(c) SCOP2: Family

**Figure S6**: Pie chart displaying the fraction of the modeled proteome associated with (a)Fold, (b)Superfamily, and (c)Family (derived from the SCOP2 database).

**Figure S7**: Front-end of CAcnesDB showcasing different functionalities.
